# Circovirus Hepatitis Infection in Heart-Lung Transplant Patient, France

**DOI:** 10.3201/eid2902.221468

**Published:** 2023-02

**Authors:** Philippe Pérot, Jacques Fourgeaud, Claire Rouzaud, Béatrice Regnault, Nicolas Da Rocha, Hélène Fontaine, Jérôme Le Pavec, Samuel Dolidon, Margaux Garzaro, Delphine Chrétien, Guillaume Morcrette, Thierry Jo Molina, Agnès Ferroni, Marianne Leruez-Ville, Olivier Lortholary, Anne Jamet, Marc Eloit

**Affiliations:** Institut Pasteur Pathogen Discovery Laboratory, Paris, France (P. Pérot, B. Regnault, N. Da Rocha, D. Chrétien, M. Eloit);; The OIE Collaborating Center for the Detection and Identification in Humans of Emerging Animal Pathogens, Paris (P. Pérot, B. Regnault, N. Da Rocha, D. Chrétien, M. Eloit);; Institut Imagine, Paris (J. Fourgeaud, M. Leruez-Ville); Université Paris Cité, Paris (J. Fourgeaud, A. Jamet);; Necker-Enfants Malades Hospital, Paris (J. Fourgeaud, G. Morcrette, T.J. Molina, A. Ferroni, M. Leruez-Ville, A. Jamet);; Hôpital Necker Enfants-Malades Centre d'Infectiologie Necker-Pasteur, Paris (C. Rouzaud, M. Garzaro, O. Lortholary);; Groupe Hospitalier Paris Saint Joseph-Marie Lannelongue, Équipe Mobile de Microbiologie Clinique, Paris (C. Rouzaud);; Hôpital Cochin Département d'Hépatologie-Addictologie, Paris (H. Fontaine);; Université Paris–Sud, Paris (J. Le Pavec);; Hôpital Marie Lannelongue Service de Pneumologie et Transplantation Pulmonaire, Le Plessis-Robinson, France (J. Le Pavec, S. Dolidon);; Institut Necker Enfants Malades, Paris (A. Jamet);; Ecole Nationale Vétérinaire d’Alfort, Maisons-Alfort, France (M. Eloit)

**Keywords:** hepatitis, circoviruses, solid organ transplants, metagenomic next-generation sequencing, mNGS, viruses, zoonoses, France

## Abstract

In March 2022, a 61-year-old woman in France who had received a heart-lung transplant sought treatment with chronic hepatitis mainly characterized by increased liver enzymes. After ruling out common etiologies, we used metagenomic next-generation sequencing to analyze a liver biopsy sample and identified an unknown species of circovirus, tentatively named human circovirus 1 (HCirV-1). We found no other viral or bacterial sequences. HCirV-1 shared 70% amino acid identity with the closest known viral sequences. The viral genome was undetectable in blood samples from 2017–2019, then became detectable at low levels in September 2020 and peaked at very high titers (10^10^ genome copies/mL) in January 2022. In March 2022, we found >10^8^ genome copies/g or mL in the liver and blood, concomitant with hepatic cytolysis. We detected HCirV-1 transcripts in 2% of hepatocytes, demonstrating viral replication and supporting the role of HCirV-1 in liver damage.

Solid organ transplant recipients are highly susceptible to infections that sometimes have uncommon clinical manifestations (*1*–*3*). Etiologic diagnosis of hepatitis is even more problematic in organ transplant recipients because a wide range of possible drug toxicities induced by immunosuppressive therapies must be considered (*4*). Diagnosing unexplained hepatitis remains a challenge, as exemplified by 74 cases of acute hepatitis of unknown etiology reported in children in the United Kingdom, 3 cases in Spain, and up to 5 possible cases in Ireland (*5*). Among transplant recipients, it is especially critical to detect infection, often viral, to prevent unnecessary interruption of treatment. Cases of direct viral transmission from the graft itself have been reported (*6*,*7*). Agnostic metagenomic analysis of clinical samples based on next-generation sequencing (NGS) combined with specifically designed PCRs constitute promising tools for diagnosing infection with unforeseen or novel microorganisms (*8*). We report identifying a novel circovirus infecting humans, tentatively named human circovirus type 1 (HCirV-1), and its role in causing liver damage in a solid organ transplant recipient.

## Case Report

**Figure 1:**
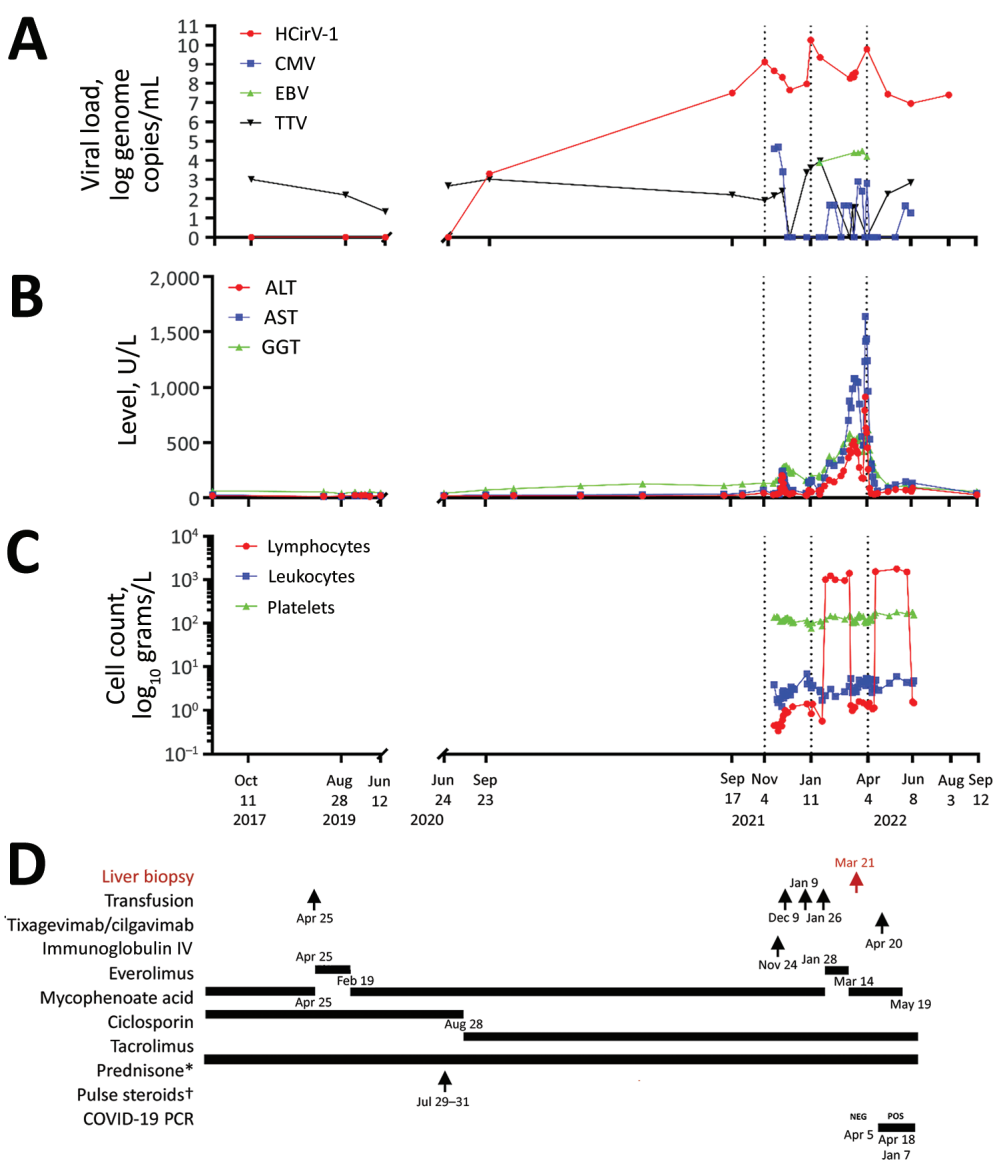
Clinical and laboratory data over time for a heart-lung transplant patient in France who had cytolytic hepatitis caused by HCirV-1 develop. A–C) Monitoring of patient over time: A) viral loads; B) liver cytolysis markers; C) blood cell counts. D) Patient’s treatment history; red indicates timing of liver biopsy (March 21, 2022). Average values are depicted for HCirV-1 and TTV viral loads in plasma and blood on June 8 and August 3, 2022. *Dose 5mg/day; †solumedrol dose 500 mg/day. ALT, alanine transaminase; AST, aspartate aminotransferase; CMV, cytomegalovirus; EBV, Epstein-Barr virus; GGT, gamma-glutamyl transferase; HCirV-1, human circovirus type 1; IV, intravenous; TTV, Torque teno virus.

A woman, 61 years of age, sought treatment in France with cytolytic hepatitis of unknown etiology. The patient had received a heart-lung transplant 17 years earlier because of Eisenmenger syndrome related to ventricular septal defect. Her maintenance immunosuppressive regimen included tacrolimus, mycophenolate mofetil, and prednisone. She had received pulse steroids 16 months before the hepatitis occurred to treat acute lung allograft dysfunction. The patient had had a localized left upper lobe pulmonary adenocarcinoma treated by surgery 2.5 years before this episode. She also had a history of chronic renal failure from calcineurin inhibitor (Cockcroft-Gault up to 23 mL/min), *Aspergillus* spp. and *Scedosporium* spp. bronchopulmonary infections, and parvovirus B19 and COVID-19 infections. In November 2021, 14 months after receiving pulsed corticosteroid therapy, the patient was hospitalized for cytomegalovirus (CMV) colitis with ganciclovir-resistant virus requiring treatment with foscavir, then maribavir, followed by letermovir for long-term maintenance therapy. At that time ultrasound examination detected cytolysis without symptoms or liver abnormalities (Figure 1).

Four months after that hospitalization, hepatitis worsened; transaminase serum levels increased to up to 40 times the normal upper limit (Figure 1), leading to hospital readmission. The patient was asymptomatic except for recent weight loss but without biological severity because the prothrombin time level was normal. We ruled out common infections, including hepatitis A, B, C, and E, as well as HIV, CMV, herpes simplex virus, varicella zoster virus (VZV), human herpes virus 6 (HHV6), adenovirus, enterovirus in blood and feces, parvovirus B19, toxoplasmosis, syphilis, and leptospirosis. Only Epstein-Barr virus (EBV) PCR was positive (10^4.4^ genome copies/mL of blood). There was no evidence for a timeline relationship between levels of liver enzymes and use of various medications by the patient. She reported no use of paracetamol (acetaminophen), alcohol, illicit drugs, or phytotherapy (use of herbal/plant supplements). A cardiac ultrasound did not show cardiac failure. We found no cardiac arrhythmia or vascular or biliary abnormalities from liver ultrasound and Doppler examinations, computerized tomography scan, or biliary magnetic resonance imaging results. Test results for autoimmune abnormalities were negative. Finally, aceruleoplasminemia test results were normal. 

**Figure 2:**
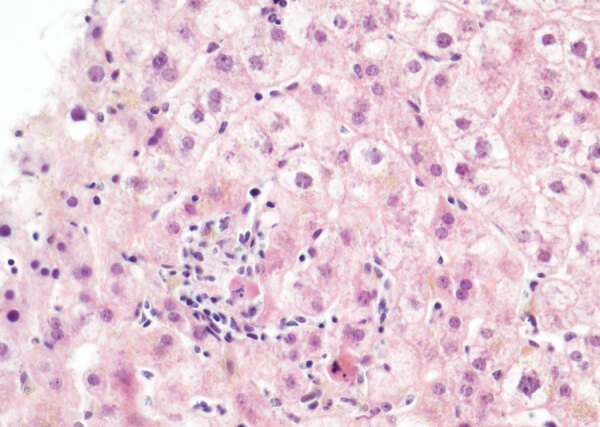
Lobular hepatitis in a heart-lung transplant patient in France. Apoptotic bodies, hepatocyte swelling and ballooning were present surrounded by a slight inflammatory infiltrate made of lymphocytes and histiocytes. Hematoxylin and eosin stain; original magnification ×40.

Pathological examination of a liver biopsy showed lobular hepatitis, slightly inflammatory, without epithelioid granuloma, substantial portal inflammation and fibrosis, or lymphoproliferation (Figure 2). Results of Ziehl, EBV, CMV, and adenovirus stains and bacteriological examinations of the biopsy sample, including mycobacterial cultures and PCR, broad-range 16S ribosomal RNA gene PCR, were negative. Herpes simplex virus 1 and 2, CMV, adenovirus, and enterovirus PCR results were negative; EBV PCR was positive (135 genome copies/μg DNA, cycle threshold = 35) but not ascribed to acute hepatitis. Overall, the lymphocytic pattern of inflammation in contrast to the lack of eosinophil infiltration, vascular or biliary injuries, and autoimmune liver disease (no plasmocytes), strongly suggested viral hepatitis. We considered the extent of both centrilobular necrosis and resorption by macrophages, which suggested recent liver cytolysis. After these first-line negative results from the liver biopsy, we conducted metagenomic NGS (mNGS), which revealed presence of the novel circovirus, HCirV-1. We further explored and quantified HCirV-1 using quantitative PCR (qPCR) on liver tissue, historical blood samples, feces, bronchoalveolar lavage, urine, and saliva samples from the patient (Table).

**Table:** Samples tested by qPCR to detect and quantify human circovirus type 1 infection in a heart-lung transplant patient in France*

Sample type	Sampling date	Ct values	Virus copies†
Serum	2017 Oct 11	Negative	Negative
Serum	2019 Aug 28	Negative	Negative
Serum	2020 Jun 12	Negative	Negative
Serum	2020 Jul 24	Negative	Negative
Plasma	2020 Sep 17	17.9	3.24 × 10^7^
Serum	2020 Sep 23	32.4	2.00 × 10^3^
Serum	2021 Nov 05	12.4	1.25 × 10^9^
Serum	2021 Nov 18	13.9	4.38 × 10^8^
Feces	2021 Nov 19	23.5	3.12 × 10^5^
Serum	2021 Nov 30	15.1	2.07 × 10^8^
Serum	2021 Dec 10	17.4	4.49 × 10^7^
Serum	2022 Jan 05	16.3	9.19 × 10^7^
BAL	2022 Jan 06	24.8	3.24 × 10^5^
Serum	2022 Jan 12	8.4	1.72 × 10^10^
Serum	2022 Jan 25	11.5	2.19 × 10^9^
Feces	2022 Jan 27	25.5	7.98 × 10^4^
Plasma	2022 Mar 10	15.3	1.77 × 10^8^
Serum	2022 Mar 14	14.6	2.77 × 10^8^
Plasma	2022 Mar 16	15.1	2.08 × 10^8^
Serum	2022 Mar 18	14.3	3.52 × 10^8^
Liver biopsy	2022 Mar 21	16.4	3.57 × 10^8^
Feces	2022 Apr 01	23.7	2.68 × 10^5^
Blood	2022 Apr 04	10.0	5.81 × 10^9^
Serum	2022 May 05	18.1	2.73 × 10^7^
Serum	2022 May 05	17.2	4.92 × 10^7^
Feces	2022 May 11	28.6	1.06 × 10^4^
Urine	2022 Jun 08	26.4	1.13 × 10^5^
Saliva	2022 Jun 08	26.0	1.45 × 10^5^
Plasma	2022 Jun 08	19.2	1.36 × 10^7^
Blood	2022 Jun 08	20.3	6.36 × 10^6^
Feces	2022 Jun 09	27.2	2.60 × 10^4^
Blood	2022 Aug 03	18.8	1.70 × 10^7^
Plasma	2022 Aug 03	17.5	4.01 × 10^7^

*Virus copies are per gram for liver biopsy and feces samples, per milliliter for all other samples. BAL, bronchoalveolar lavage; qPCR, quantitative PCR; Ct, cycle threshold.

In November 2022, the patient’s clinical condition was stable, and we noted complete recovery from cytolysis. In the context of her recent infectious diseases, we minimized the patient’s immunosuppressive treatment, discontinuing mycophenolate mofetil (Figure 1). When we tried to ascertain possible sources of her HCirV-1 infection, the patient reported contact with 2 cats, both of which had contact with birds and rodents. HCirV-1 PCR was negative in the feces of the cats 2 months after her diagnosis. The patient reported no international travel. She did report receiving a blood transfusion 17 months before HCirV-1 was first detected (Figure 1).

## Methods

### Description of Controls

We performed retrospective HCirV-1 qPCRs on feces and blood samples from immunocompromised or immunocompetent control patients with or without hepatitis (Appendix Table 1). We also reviewed the results of mNGS routinely performed since 2019 on all liver biopsies in our laboratory to detect pathogens (Appendix Table 2).

### Nucleic Acid Extraction

We extracted nucleic acids from the patient’s liver biopsy using the geneLEAD VIII instrument (Diagenode, https://www.diagenode.com). We extracted nucleic acids from 200 μL of plasma, serum, whole blood, bronchoalveolar lavage (BAL), and feces using the bioMérieux Emag instrument (https://www.biomerieux.com).

### mNGS

We generated complementary DNA from a biopsy from the patient’s liver using the Invitrogen SuperScript IV First-Strand Synthesis System kit (ThermoFisher Scientific, https://www.thermofisher.com) and used MALBAC amplification (Yikon Genomics, https://en.yikongenomics.com) as described elsewhere (*9*) to use as input for Illumina DNAPrep (https://www.illumina.com). We sequenced the library in 1 × 151 bp on an Illumina NextSeq500 instrument generating ≈80 million raw reads. We processed raw reads with Microseek (*10*) using the RVDB-prot reference viral database (*11*).

### qPCR HCirV-1

We designed PCR primers HCirV1-Fw1, 5′-ACCTGGATGGACCCTGGAAT-3′, and HCirV1-Rv1, 5′-AGAGTTCCACCAGGTTCTGC-3′ (194 bp), in the capsid gene of HCirV-1. We performed qPCRs in SYBR green format with 45 cycles of amplification at an annealing temperature of 58°C and purified positive amplicons after 45 cycles on gel, confirmed them by Sanger sequencing, and used them for serial-dilutions to generate standard curves for the calculation of viral loads.

### Acquisition of Complete Genome

After preamplification with the QIAGEN whole transcriptome analysis ϕ29 polymerase (https://www.qiagen.com), we obtained the full genome of HCirV-1 by sequencing plasma from the patient, sampled on January 12, 2022, which harbored 10^10^ viral copies/mL. We applied an iterative mapping approach using Geneious Prime 2022.1.1 (https://www.geneious.com) to perform the final assembly and deposited the complete genome sequence at GenBank (accession no. ON677309).

### Phylogeny

**Figure 3:**
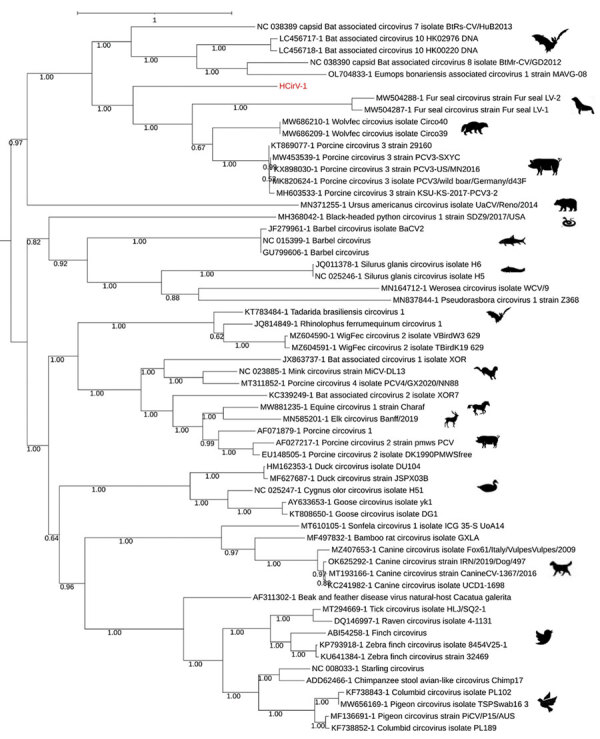
Phylogenetic analysis of capsid protein sequences of human circovirus type 1 (HCirV-1) from a heart-lung transplant patient in France (red) and representative circovirus strains. Sequences were aligned with Multiple Alignment using Fast Fourier Transform (https://www.ebi.ac.uk/Tools/msa/mafft) under the L-INS-I parameter, and maximum-likelihood phylogenetic reconstruction was performed with PhyML implemented through the NGPhylogeny portal (https://ngphylogeny.fr). GenBank accession numbers for reference sequences are indicated, along with graphic representations of the animal of origin. Scale bar indicates the number of amino acid substitutions per site.

We performed phylogenetic reconstructions on the capsid protein sequence (Figure 3), on the replicase (Rep) protein sequence (Appendix Figure 1), and on the complete nucleotide sequence of HCirV-1 (Appendix Figure 2). We aligned complete open reading frames of the capsid and Rep genes along with other representative sequences of circoviruses using Multiple Alignment using Fast Fourier Transform (https://www.ebi.ac.uk/Tools/msa/mafft) under the L-INS-i parameter. We performed maximum-likelihood phylogenetic reconstruction with PhyML implemented through the NGPhylogeny portal (*12*) and evaluated nodal support using the approximate Bayes branch supports. 

### TTV and EBV qPCR

We performed TTV (Torque teno virus) DNA load using the TTV R-GENE kit (bioMérieux, https://www.biomerieux.com) as described elsewhere (*13*) and EBV DNA load using the bioMérieux EBV R-GENE kit. We performed real-time PCR amplification on an Applied Biosystems AB7500 platform (ThermoFisher).

### In Situ Hybridization

We used a ThermoFisher 2-plex ViewRNA ISH Tissue Assay Kit for in situ hybridization (ISH) to detect RNA sequences of HCirV-1 as a marker of virus replication. We designed a cocktail of 12 custom-branched DNA probes to target HCirV-1 capsid genes and 20 probes to target Rep genes, which were revealed by a red signal (probe type 1 in the assay kit); a blue signal (probe type 6 in the kit) revealed a mix of control probes targeting human housekeeping transcripts. We applied 20-min heat pretreatment and 15-min protease digestion to tissue sections after deparaffinization using xylene to unmask RNA targets and used Harris hematoxylin for counterstaining. We used negative controls for ISH that included a no-probe negative control from the patient infected with HCiV-1, as recommended by the manufacturer, and a negative control biopsy sample from a different patient that tested negative for HCirV-1 with mNGS.

We obtained informed consent for publication of our findings from the patient. The samples we analyzed were from control patients in a retrospective noninterventional study with no additional standard care procedures. Our study was performed in accordance with ethical guidelines for medical research and approved by the institutional review boards of Necker Hospital.

## Results

### Identifying Distant Circovirus Sequences by Liver Biopsy

Using NGS to analyze viral sequences from the liver biopsy specimen, we identified 1,011 sequences corresponding to the capsid and Rep genes from the *Circoviridae* family. From a plasma sample taken at the peak of viremia, we derived a consensus full-genomic sequence (2,021 nt) of the HCirV-1 virus (Genbank accession no. ON677309) and confirmed that the sequence was identical to partial sequences of the capsid gene found in the liver. We conducted phylogenetic analyses on the 2 HCirV-1 viral proteins, capsid and Rep, and on representative animal circoviruses; congruent analyses showed that HCirV-1 defines a new clade at a basal position relative to its closest viral species, Wolvfec circovirus (host: *Gulo gulo*) and porcine circovirus 3.

### Kinetics of Infection

We designed an assay and conducted qPCR to assess changes in the viral load in plasma and other biologic samples taken from the case-patient since 2017. We also evaluated the viral load for TTV, a commensal virus whose level of viremia is well correlated with the immune status of graft recipients (*14*). TTV DNA load varied (0–3.99 log copies/mL), indicating a low level of immune suppression (*15*); peaks in TTV viremia and cytolysis were not temporally correlated. In contrast, HCirV-1 was first detected in patient blood samples (10^3.3^ genome copies/mL) in September 2020, 2 months after steroids pulse therapy, but a July 2020 sample tested negative. However, it is noteworthy that the finding of a low level of HCirV-1 in the September 2020 sample was questionable because the tubes were opened at the same time as others harboring up to 10^9.1^ genome copies/mL, possibly resulting in cross-contamination. The next available sample, taken in September 2021, was strongly positive (10^7.5^ genome copies/mL); thereafter, loads varied (10^8.3^–10^10.2^ genome copies/mL). HCirV-1 loads were high only during the period of pronounced cytolysis.

### Assessing HCirV-1 Presence in Controls

We tested 36 blood and 20 feces samples from 56 control patients using the same specific qPCR primers for assessing HCirV-1 presence, but none tested positive (Appendix Table 1). Among those patients, 19 (34%) were primitively immunocompromised, 17 (30%) immunocompetent, 13 (23%) solid organ transplant recipients, 5 (9%) hematopoietic stem cell transplant recipients or receiving immunosuppressive treatment, and 2 (4%) HIV positive. Among 16 patients with hepatic cytolysis (Appendix Table 1), 8 cases were of unknown etiology. Control biopsies routinely screened by mNGS since 2019 did not show HCirV-1 sequences among 57 patients with hepatitis of suspected infectious origin, among whom 25 (44%) were primitively immunocompromised, 20 (35%) hematopoietic stem cell transplant recipients or receiving immunosuppressive treatment, 5 (9%) immunocompetent, 5 (9%) solid organ transplant recipients, and 2 (4%) HIV positive (Appendix Table 2).

### ISH

**Figure 4:**
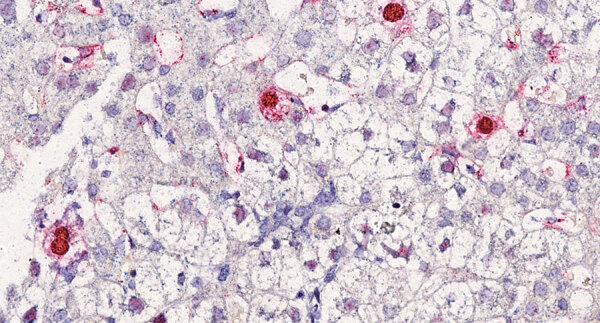
In situ hybridization in liver section from a heart-lung transplant patient in France. Chromogenic in situ hybridization detected human circovirus type 1 mRNA (red staining) in hepatocytes nuclei and cytoplasm. Nuclei were counterstained with Harris hematoxylin and eosin stain; original magnification ×40.

ISH of the liver biopsy specimen showed 2% infected hepatocytes (Figure 4); red labeling was strong in the nuclei and mild in the cytoplasm. Some hepatocytes were also poorly labeled in the nuclei, and, in this case, labeling was negative in the cytoplasm. Endothelial cells and lymphocytes were not infected. No red signal was recorded in the ISH-negative controls. 

## Discussion

Our findings show a temporal association between hepatitis and a high load of a novel virus, HCirV-1, in the liver and blood of a 61 year-old female heart-lung transplant recipient. The patient received standard immunosuppressive therapy of tacrolimus, mycophenolate mofetil, and corticosteroids, but CMV colitis developed a year after pulsed corticosteroid therapy, suggesting a contemporary drug-induced high level of immunosuppression. Our results should be evaluated with Hill’s criteria for causality (*16*).

We demonstrated the specificity of association using agnostic mNGS in the liver biopsy sample, showing absence of any additional virus except PCR-detected EBV. Given the low concentration of EBV in the liver biopsy, the concomitant higher replication in the blood (10^4.4^ genome copies/mL), and the negative result from staining on liver biopsy, we did not consider EBV to be responsible for the cytolysis. In total, we did not detect HCirV-1 in 113 control participants: 40 tested by qPCR without hepatitis, 16 with hepatitis of known (50%) or unknown (50%) etiology (Appendix Table 1), and 57 mNGS-tested case-patients with hepatitis of unknown etiology (unpub. data, Appendix Table 2).

Circoviruses are DNA viruses. Of note, transcripts were detected in the nucleus of 2% of liver cells by HCirV-1–specific ISH, therefore consistent with viral expression and probably with virus replication. Given the lytic cycle of circoviruses, the results strongly support the role of HCirV-1 as the causative agent of hepatitis by targeting and lysing hepatocytes and also likely as a trigger of immune cytotoxic responses.

Animal circoviruses, such as porcine circoviruses (PCV) 1–4, have been responsible for reproductive failure, dermatitis, nephropathy, and respiratory diseases in animals (*17*,*18*), manifestations not found in the case we report. However, PCV2 associated with cases of jaundice in pigs (*19*–*21*) and hepatitis in a horse have been demonstrated (*22*). After PCV3 is inoculated into piglets, viremia progressively increases up to 10^8.9^ copies/mL, reflective of findings in our study, but generally peaks around 21 days after infection, contrary to the more delayed peak we identified. Pathological lesions and PCV3 antigens have been detected in the liver, including white-gray nodules and necrosis with PCV3 antigen detected in liver lobular stroma, the hepatic sinus antral wall, and the cytoplasm in the sinus (*23*) and in other organs (lung, heart, kidney, lymph nodes, spleen, and small intestine) (*24*). PCV3 viremia could still be observed 140 days after infection (*25*), which is indicative of the ability of circoviruses to sustain long-term infection, as described in our patient. PCV3 was also able to infect nonhuman primate recipients of porcine cardiac xenografts (*26*,*27*).

Supporting the plausibility of HCirV-1 as disease agent, we found no temporal link for drug etiology or pathological evidence as alternative explanations. We ruled out common hepatitis virus infections, including hepatitis E virus. EBV test results were positive, but we ruled out a pathogenic role because EBV viral load remained stable around 10^4^ genome copies/mL (Figure 1) and Epstein-Barr encoding region (EBER) staining was negative. 

We first detected HCirV-1 before the liver pathology testing. Viral loads peaked 3 times, before the first and second cytolysis peaks and a third time concurrently with cytolysis peak (Figure 1). The high viral load of HCirV-1 (10^10^ genome copies/mL) also favors interpretation of its causative role in hepatitis. Indeed, a strong relationship between high virus load and disease severity has been described for hepatitis A (*23*) and B (*28*) viruses and adenovirus in hematopoietic stem cell transplant recipients (*29*), further illustrating that viral load often influences organ pathology (*30*). 

As of November 2022, we had not identified the source of infection for this patient; specifically, we were unable to determine whether it was because of spillover of an animal virus or if it might be a previously unknown human virus. Although phylogenetic analysis placed this virus closest to known viruses hosted by a wild exotic animal that was not a possible source for our patient, we speculate that that HCirV-1 could be of animal origin, possibly from a dietary source in a similar manner as hepatitis E virus. Developing a specific antibody test will be instrumental in deciphering the ecology of the virus and better understanding its mechanisms of transmission. HCirV-1 was shed in saliva, urine, and feces (Table); whether the virus can possibly be transmitted between persons remains to be investigated.

In conclusion, detection of HCirV-1 expression in liver cells by ISH, the exclusion of other hepatitis etiologies, and the temporal association of severe cytolysis with very high HCirV-1 loads in the liver and blood strongly suggest a causal link. Furthermore, our experience using mNGS in immunocompromised patients, including transplant recipients, as well as the specific qPCR we performed on a representative cohort of these patients prove that, unlike TTV, HCirV-1 is not part of the commensal viral flora. Agnostic NGS was key to elucidate the etiology of this hepatitis case. HCirV-1 could now be tested for in hepatitis cases of unknown origin in solid organ transplant recipients.

Appendix Additional information about circovirus hepatitis infection in heart-lung transplant patient in France.
