# Supplementary material for: Circovirus Hepatitis Infection in Heart-Lung Transplant Patient, France
Source: Emerg Infect Dis. 2023 Feb;29(2):286–93. doi: 10.3201/eid2902.221468 (PMC9881760; doi:10.3201/eid2902.221468)
Supplement: Appendix — Additional information about circovirus hepatitis infection in heart-lung transplant patient in France. [file 22-1468-Techapp-s1.pdf]

Article DOI: https://doi.org/10.3201/eid2902.221468

# Circovirus Hepatitis Infection in Heart-Lung Transplant Patient, France

## Appendix

**Appendix Table 1.** Control characteristics for assessment of human circovirus type 1 (HCirV-1) presence in 36 blood and 20 stool samples performed with specific HCirV-1 qPCR*

| Controls | Hepatitis†(n=16) | Immunocompetent, no hepatitis (n=12) | Immunocompromised, no hepatitis (n=28) | Total (n = 56) |
|---|---|---|---|---|
| Age, mean (range), y | 17 (3–53) | 30 (0.2–67) | 44 (1–84) | 33 (0.2–84) |
| Adults >15 years old, n (%) | 5 (31) | 7 (58) | 22 (78) | 35 (63) |
| Sex, female, n (%) | 4 (25) | 7 (58) | 11 (39) | 22 (39) |
| Samples tested, n (%) | | | | |
| Blood | 10 (62) | 8 (66) | 18 (64) | 36 (64) |
| Stool | 6 (38) | NA | NA | NA |
| Known hepatitis etiology‡ | 8 (50) | NA | NA | NA |
| Immunological status, n (%) | | | | |
| Immunocompetent | 5 (31) | 12 (100) | 0 | 17 (30) |
| HIV seropositive | 0 | 0 | 2 (7) | 2 (4) |
| Solid organ transplant | 1 (6) | 0 | 12 (43) | 13 (23) |
| Primary immune deficiency | 10 (63) | 0 | 9 (32) | 19 (34) |
| HSCT or immunosuppressive therapy | 0 | 0 | 5 (18) | 5 (9) |

*HSCT, hematopoietic stem cell transplant; NA, not applicable
†hepatomegaly or liver cytolysis
‡Infectious (4 viral, 1 bacterial), dysimmune (1), metabolic (1) and toxic (1) etiologies

**Appendix Table 2.** Control characteristics for assessment of human circovirus type 1 (HCirV-1) presence in 57 liver biopsies samples screened with metagenomic next generation sequencing

| Controls | Total (n=57) |
|---|---|
| Age, y, mean (range) | 13 (0.3–68) |
| Adults >15 y, n (%) | 10 (18) |
| Sex, female, n (%) | 20 (35) |
| Immunological status, n (%) | |
| Immunocompetent | 5 (9) |
| HIV seropositive | 2 (4) |
| Solid organ transplant | 5 (9) |
| Primary immune deficiency | 25 (44) |
| HSCT or immunosuppressive therapy | 20 (35) |

*HSCT, hematopoietic stem cell transplant

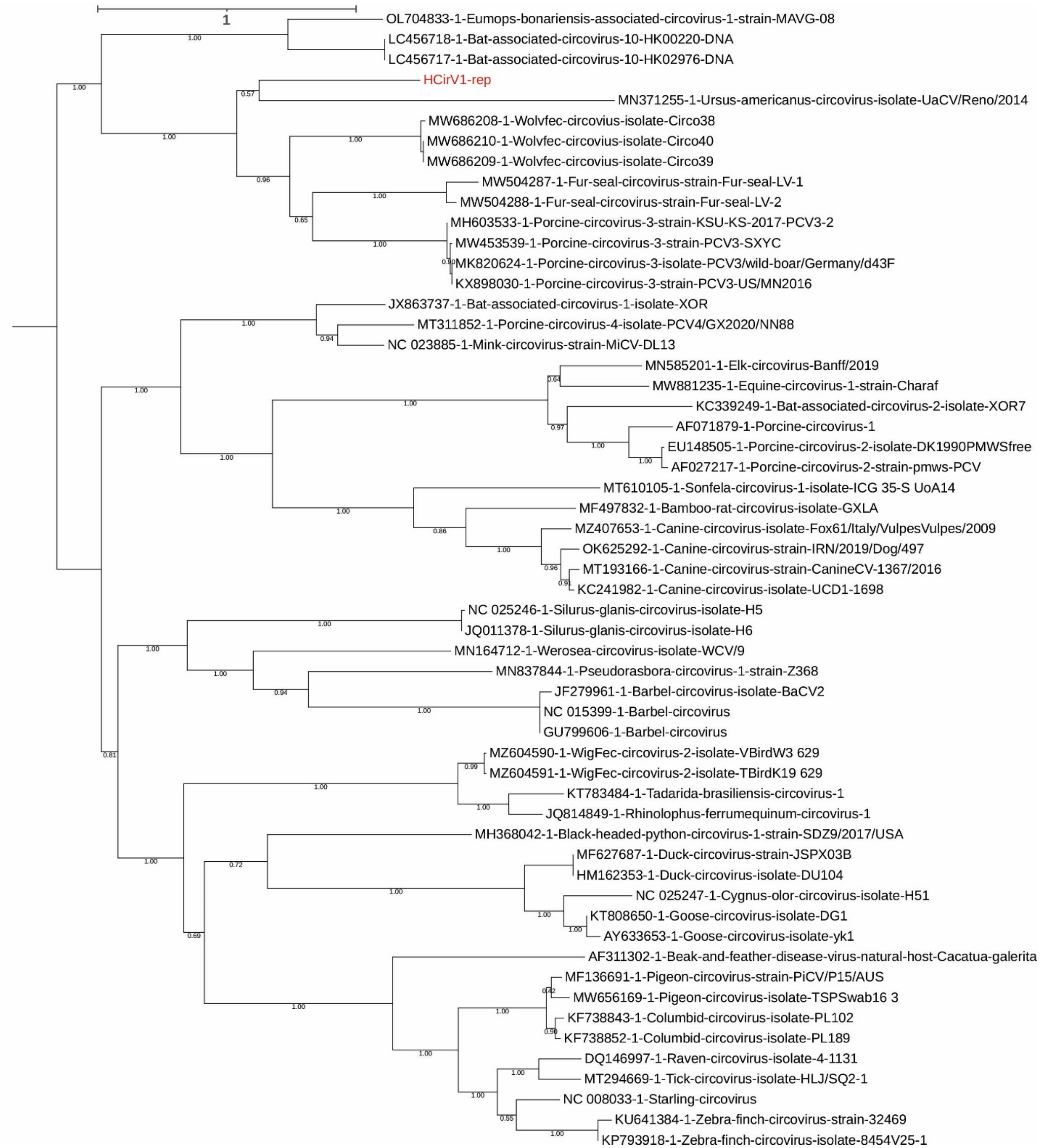


**Appendix Figure 1.** Phylogenetic analysis of human circovirus type 1 (HCirV-1). Phylogenetic analysis of replicase (Rep) protein sequences of HCirV-1 and representative of circovirus strains. Sequences were aligned with MAFFT under the L-INS-I parameter, and maximum-likelihood phylogenetic reconstruction was performed with PhyML implemented through the NGPhylogeny portal. HCirV-1 protein sequence is depicted in red. The tree scale indicates the number of amino acid substitutions per site.

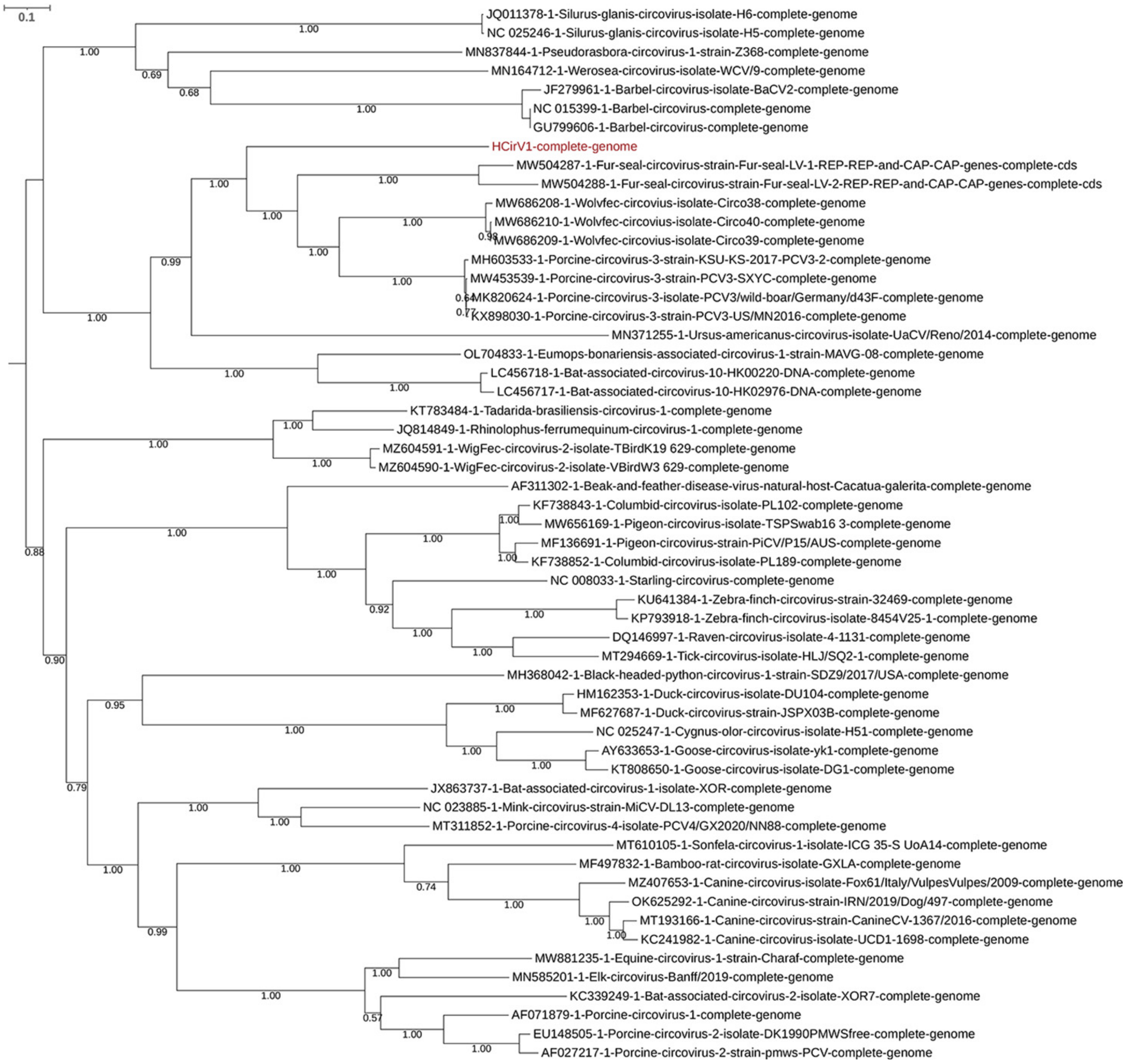


**Appendix Figure 2.** Phylogenetic analysis of human circovirus type 1 (HCirV-1). Phylogenetic analysis of complete nucleotide sequences of HCirV-1 and representative of circovirus strains. Sequences were aligned with MAFFT under the L-INS-I parameter, and maximum-likelihood phylogenetic reconstruction was performed with PhyML implemented through the NGPhylogeny portal. HCirV-1 complete genome is depicted in red. The tree scale indicates the number of amino acid substitutions per site.
